# Letter to Dr. Ramsay, in Consequence of His Observations on the Bite of a Snake Cured by Volatile Alkali

**Published:** 1804-04-01

**Authors:** Benjamin Barton

**Affiliations:** Philadelphia


### Letter *to* Dr. Ramsay, *in Consequence of his* Observations *on the* Bite *of a* Snake *cured by* Volatile Alkali. *By* Benjamin Barton, M.D. *of Philadelphia.*

Dear Sir,

I Have seen your Observations on "the Bite of a Snake cured by Volatile Alkali." I rejoice to find that you have undertaken the investigation of a subject so important as is the one on which you have favoured the public with the result of your experience. I hope you will pursue the inquiry, as I am well persuaded that we are in want of something much more efficacious as a cure of the evil effects induced by the venom of the rattlesnake, and other serpents, than any of the many vegetables which have been recommended to the public in such extravagant and unqualified terms.

Ever since the spring of 1801, I have paid a great deal of attention to the effects of the venom of the rattlesnake. I have had a number of these reptiles, both old and young, under my immediate care, and have caused them to bite many and various species of animals, with the view to collect materials for a history of the poison. My experiments have satisfied me, that the venom of the rattlesnake is one of the most deleterious substances with which we are acquainted. In many instances, the effects of the poison were observed *almost instantaneously*; and so rapid is the progress of effects, that several of the bitten animals, such as rabbits, dogs, &c. died in about thirty minutes. I may add, that a few weeks ago a man died in Jersey in twenty-seven minutes after he had been bitten by a rattlesnake.

The infirm state of my health, which compelled me to leave the city last summer, prevented me from pursuing my experiments as I had wished to have done; and the death of of the only remaining two of my snakes, by the cold of the succeeding winter, has put it out of my power to do any thing on the subject this summer. But I shall not neglect to resume the inquiry next spring, as I have the promise of a number of living snakes; and I shall take great pleasure in making you early acquainted with the result of my experiments.

One of the objects of my inquiry is the discovery of the best means of preventing or of curing the disease occasioned by the bite of the rattlesnake, and others of our venomous serpents, such as the copper-head, &c. I feel no disposition to exhaust any of my time in experimenting with all, or even a twentieth part, of the many vegetables which have been praised and employed for these purposes in different parts of the United States. Many of these are unquestionably inert, and I think I have elsewhere shown how they have acquired their reputation. I am far from denying that some of the vegetables to which I allude are deserving of a *portion* of the praise which has been bestowed upon them. The seneca snake-root (*polygala senega* of Linnæus) is, without doubt, a plant of great powers, and may be worthy of our attention as a remedy against the bite of venomous serpents. You know that among the Indians this plant has sustained a high reputation in this respect. One of my correspondents (Mr. Samuel Preston, of this state,) has communicated to me a case which is worthy of being mentioned. In the year 1798, a man, whilst he was mowing, was bitten by a rattlesnake in the little toe of his foot. Almost instantly he was seized with a pain in his breast and eyes. The leg became greatly swollen, and violent symptoms of a genuine tetanus ensued. The seneca, which was at hand, was boiled in milk, and the patient drank large quantities of the decoction, at the same time that the root, in the shape of a poultice, was applied to the part immediately wounded. The medicine threw him into a profuse perspiration; in a short time all his spasms subsided, and at the end of two days he was able to return to his occupation of mowing again.

This case, to which I have alluded in my *Elements of Botany*, part iii. p. 105, is certainly an important one, as I think it plainly shows that the seneca is a medicine well adapted to *some* cases of the bite of a rattlesnake. I rather regret that, in the work just mentioned, I should have used the following words, when speaking of the medicine:—" Its great virtues as a remedy for the bite of the rattle-snake may, I believe, be safely called in question." I am

 far, far, indeed, from supposing that it is an infallible medicine or specific: on the contrary, I have not a doubt that it would fail to effect a cure in many cases of the bites of venomous serpents. What relief could we expect from it, or indeed from any thing else, in cases (such as the one in Jersey) where death was induced in less than half an hour?

I shall not omit to try the effects of the volatile alkali. Hitherto I have not used it, because I had imagined that correct experiments had nearly robbed it of all its former reputation. It appears from the Abbé Fontana's highly interesting work on *poisons*, that this alkali, whether externally applied or internally exhibited, was of no use in diminishing the activity of the venom of the viper, which is so very similar to that of our rattlesnake. One quotation from the Italian philosopher's work I beg leave to lay before you; "I had (he says) several animals, such as hens, rabbits, guinea pigs, &c. bit in the leg, and some minutes after made deep and extensive incisions into the wounded parts. I washed these incisions with pure volatile alkali, and covered the legs with linen bandages. I got ready an equal number of animals of the same size, and of the same kinds, to serve as a comparison. These were likewise bit in the leg; but I neither made incisions, nor applied to them the volatile alkali. The result of twenty-four experiments was not favourable to this medicine applied to the incisions, and the violence of the disease was even more considerable in the former than in the latter. Upon the whole, Fontana is of opinion that his experiments "not only demonstrate the absolute inutility of the volatile alkali against the bite of the viper, when applied externally; but," that "they at the same time prove still farther that t cannot have a direct and specific operation when it is even taken internally."

I do not pretend, Sir, to decide between your experience and the experiments of Fontana. I can, in great sincerity, assure you that I repose much confidence in your caution and accuracy in conducting medical inquiries, and in your candour in relating your observations. But do we not very often describe effects, both good and bad, to our remedies, which those remedies have not produced? Do not our patients sometimes recover from violent diseases without the aid of any medicines whatever? Does not Nature (that is, the powers or tendencies of the constitution) very frequently cure a gonorrhœa? Nay, do we not find the same powers, in some instances, sufficient to cure the malignant disease of yellow fever?

The

The result of all my inquiries relative to the poison of serpents is very favourable to the opinion, that the instances of spontaneous recovery from the influence of these poisons are numerous. Without admitting this position as a fact, what satisfactory explanation can be offered of the many recoveries from the bite of the rattlesnake, when the vegetables that were applied externally, or exhibited internally, were, if not nearly inert (mere *nutrientia*,) at least endued with the most opposite powers? Thus the bulb of *hypoxis erecta* (which grows abundantly in Carolina and other parts of the united States) is by many persons deemed a sovereign remedy against the bite of the rattlesnake. But this bulb, which I have often eaten, is almost as mild and inert as boiled rice.

That an animal which has been bitten by a rattlesnake, whose poison has induced most violent symptoms, such as acute pain, fever, and even palsy of the extremities, shall completely recover from these symptoms without the aid of any medicine whatever, is a fact which is familiarly known to me from my own experiments.

Permit me to suggest to you the propriety of employing active emetics, so as to excite full vomiting, in some of the cases of the bites of serpents that may come under your care. The very striking analogy which subsists between the effects of the venom of the rattlesnake and those of the poison inducing malignant yellow fever, has led me to suspect that emetics might be useful in the former as well as in the latter of these cases. Fontana found the tartar emetic useful in cases of the bite of the viper; and Dr. Boys, an intelligent physician who is settled at Staunton in Virginia, informed me (in my visit to that place last summer) that he had found emetics, so managed as to excite both puking and purging, *more useful* than any thing else in removing the symptoms originating from the bite of the copper-head snake, which is not less venomous than the rattlesnake.

*July* 23, 1803.

